# Understanding psychological distress affecting surgical oncology patients during the COVID-19 pandemic—experience from a tertiary cancer care center in the Middle East

**DOI:** 10.3389/fpsyg.2025.1427835

**Published:** 2025-10-30

**Authors:** Mahmoud Al-Masri, Yasmin Safi, Mohamad K. Abou Chaar, Hani Al-Najjar, Sobuh Abu Shanab, Khawlah Ammar, Amani Manfalouty, Basim Aljalabneh

**Affiliations:** ^1^Department of Surgery, King Hussein Cancer Center, Amman, Jordan; ^2^Psychosocial Oncology Program, King Hussein Cancer Center, Amman, Jordan; ^3^Survey Unit and Research Office, King Hussein Cancer Center, Amman, Jordan; ^4^Department of Nursing, King Hussein Cancer Center, Amman, Jordan

**Keywords:** distress, pandemic, cancer, surgery, COVID-19, coronavirus, psycho-oncology, psychology

## Abstract

**Background:**

The COVID-19 pandemic brought stress and anxiety on a global level, and the rapid growth of cases forced medical facilities to redesign their services, prioritizing urgent surgical care. Cancer centers worldwide continued to function, some at limited capacity. However, many procedures were adapted to avoid unnecessary risks of exposure on staff and patients. Testing have also led to inevitable delays in surgical schedules and placed a burden on vulnerable patients who were already suffering from psychological stress. This study focused on assessing overall psychological distress in cancer patients at King Hussein Cancer Center (KHCC) who required primary curative or debulking surgery and whose operations were rescheduled due to the pandemic.

**Methods:**

A cross-sectional study was conducted between 17/April/2020 and 25/May5/2020 using an automated self-administered questionnaire. Distress levels were measured using a tailored scale that combined items from validated instruments (Taylor, HADS, and Zung).

**Results:**

A total of 264 participants completed the questionnaire. Mean age was 54 years (±14.9 years), and genders were equally represented. Breast cancer cases were the most frequent (*n* = 79, 27.65%). Patients younger than 54 years, regardless of gender (*n* = 125, 47%), and females in general (*n* = 140, 53%), scored the highest levels of distress (*p* < 0.0001, *p* = 0.018, respectively). Marital status had a positive impact on distress levels (*p* = 0.008). Central nervous malignancies were associated with higher levels of stress on the multivariate analysis (*p* = 0.004). Patients who were still experiencing a surgical delay at the time of data collection reported significantly higher distress levels compared to those who had already undergone their delayed or rescheduled surgery (*p* = 0.001).

**Conclusion:**

Based on our data, we noticed higher levels of distress in younger individuals (younger than 54 years) and in female participants. The highest levels of distress were recorded in patients awaiting treatment and in those who were diagnosed with central nervous system cancer regardless of their treatment status. Higher levels of stress were reported in COVID-19 specific questions. Our results replicate what was published before in the literature associating the COVID-19 pandemic with stress among cancer patients.

## 1 Introduction

With the emergence of SARS-Cov-2 by the end of 2019, and after confirming person-to-person transmission, and with the rapid rise in cases, the WHO declared the novel virus infection a pandemic on March 11, 2020 (Li et al., 2020). The first confirmed case of COVID-19 in Jordan was on March 2, 2020. During that time, media coverage of the exponential spread of the disease was able to deliver important instructions and information regarding the status of the pandemic. Unfortunately, a related psychological distress emerged due to continuous exposure to disease-related updates (Garfin et al., 2020), followed by the state of quarantine and self-isolation that was implemented in multiple countries, including Jordan.

A cancer diagnosis is reported to be debilitating in nature and places the patients and their families in a state of fear (Maguire et al., 1978; Wortman and Dunkel-Schetter, 1979). One of the major coping mechanisms in such vulnerable populations is information seeking, which is utilized to reduce uncertainty and decrease panic (Wiesenthal, 1984). Sadly, during the COVID-19 pandemic, such knowledge placed cancer patients in a state of continuous distress, knowing they are more susceptible than others to contract this viral illness, and that they are associated with higher mortality (Liang et al., 2020).

Psychological distress is a multidimensional construct encompassing emotional suffering—such as anxiety and depressive symptoms—as well as physical complaints like insomnia or palpitations. Among oncology patients, psychological distress is common and may negatively impact quality of life, treatment adherence, and clinical outcomes (Huda et al., 2022).

Although distress during the COVID-19 pandemic has been studied in the general population (Miaskowski et al., 2020), few investigations have focused on cancer patients—a particularly vulnerable group. Prior findings suggest increased distress among females, younger individuals, and those impacted by lockdowns, unemployment, or health-related uncertainty (Wang et al., 2020; Qiu et al., 2020; Jahanshahi et al., 2020; Zhang et al., 2021; Gómez-Salgado et al., 2020). However, research specific to oncology populations, especially those experiencing surgical delays, remains limited.

Understanding the psychological impact of such treatment disruptions may help identify key distress patterns and inform interventions in future global health emergencies. To our knowledge, this is the first study to assess distress levels among surgical oncology patients in a Middle Eastern setting during the COVID-19 pandemic. Therefore, this study aimed to assess general distress levels—including anxiety and depressive symptoms—using a composite tool that integrates items from validated instruments (Taylor, HADS, Zung). The target population included cancer patients at King Hussein Cancer Center (KHCC) who required primary curative or debulking surgery and whose operations were rescheduled due to the COVID-19 pandemic.

## 2 Materials and methods

### 2.1 Study design and data collection

This cross-sectional study was conducted at King Hussein Cancer Center (KHCC), a leading tertiary referral center for comprehensive cancer care in the Middle East. The largest tertiary referral center for comprehensive cancer care in Jordan and a leading institution in the Middle East. In 2024, KHCC registered 5,680 new cancer cases, managed 17,767 admissions, and provided active treatment to 7,544 patients. Additionally, 5,075 patients were on survival care and 13,695 were under follow-up. With its large and diverse patient base, including a substantial surgical oncology population, KHCC provides an ideal setting to evaluate the psychological impact of treatment delays and disruptions during the COVID-19 pandemic.

After acquiring an Institutional Review Board Approval (20 KHCC 72) and in order to adhere to physical distancing measures preventing SARS-Cov-2 transmission, an online Google document form (Google® 1,600 Amphitheatre Parkway, Mountain View, California, U.S.A.) was used to populate a questionnaire for collecting data between 17/4/2020 and 25/5/2020.

The study population consisted of adult patients (≥18 years) diagnosed with solid tumors who were scheduled for surgical treatment at KHCC during the early months of the COVID-19 pandemic (April–May 2020). Inclusion criteria were patients with confirmed cancer diagnoses requiring surgical excision in the operating room, and whose surgical plans were directly impacted by the COVID-19 pandemic, either by delay, modification, or rescheduling. Exclusion criteria included patients with metastatic disease, hematological malignancies, or those whose treatment plans involved interventional radiology rather than surgical intervention.

The participants were identified from both the surgical clinic logbook and backlog. At the time of data capture, a total of 324 eligible patients were screened. Of these, 40 patients were excluded due to metastatic disease (*n* = 21), hematological malignancies (*n* = 13), or receipt of interventional radiology procedures (*n* = 6). The final study sample consisted of 284 patients who met the inclusion criteria, 264 agreed to participate and completed the online questionnaire (response rate = 93%). No formal sample size calculation was performed in advance, as this was an exploratory study conducted during the early months of the pandemic, and our intent was to capture the entire cohort of eligible patients directly affected by surgical delays at KHCC during the study period. By including nearly all eligible patients, the study ensured a broad and representative overview of the target population. To maintain physical distancing and adhere to institutional COVID-19 safety protocols, all interviews were conducted remotely through telephone and online platforms; no in-person data collection was undertaken.

Patients were contacted via telephone in order to obtain verbal consent. Upon approval to participate, a link containing the address for the aforementioned questionnaire was sent using WhatsApp. During the verbal consent process, it was indicated to each individual that participation was voluntary, and we were able to provide assistance to our elderly patients with either over the-phone instructions or assisting a younger relative to help in filling the questionnaire.

### 2.2 Questionnaire and data analysis

The questionnaire collected demographic information, including age, sex, marital status, monthly income, insurance coverage, and details about any delays in treatment plans. With input from the Department of Psychosocial Oncology at KHCC, a 39-item COVID-19 Distress Scale was developed, tailored to oncology patients. The tool combined elements from three validated instruments: the Taylor Manifest Anxiety Scale, the Hospital Anxiety and Depression Scale (HADS), and the Zung Self-Rating Depression Scale (Taylor, 1953; Zigmond and Snaith, 1983; Zung, 1971). Responses were recorded on a three-point Likert scale (Always = 2, Sometimes = 1, Never = 0), ensuring ease of use and interpretability (see Appendices A and B).

The scale addresses 34 questions related to participants’ general psychological status, plus 5 questions specifically targeting the impact of the COVID-19 pandemic on their lives and daily routines (specifically items 21 and 24–27 in the questionnaire). To capture a full picture of distress, the scale also includes physical complaints, such as palpitations, headaches, and stomachaches. Additionally, statements were included to assess the effects of treatment delays on psychological status, as well as participants’ understanding of how pandemic-related measures might impact their overall health.

Distress levels were quantified by calculating a total distress score for each participant, obtained by summing responses to all 39 items on the scale, with a maximum possible score of 78. In addition, a pandemic-specific subscore was computed by summing the responses to the five COVID-19-related items, enabling the assessment of distress specifically attributable to the pandemic context.

For statistical analysis, participants were not stratified into predefined stress categories (e.g., low, moderate, or high). Instead, a continuous scoring approach was adopted. Total and subscale scores were calculated for each participant, followed by computation of the mean total distress score and mean pandemic-related subscore across various demographic and clinical subgroups, including age, gender, and cancer diagnosis. This analytical method allowed for a more flexible and representative comparison of average distress levels across groups without reliance on arbitrary score thresholds.

Before data collection, we circulated the questionnaire among experts to validate its content. A pilot study involving 10 patients was conducted to further assess the scale’s reliability and validity. Participants’ responses were analyzed, resulting in a Cronbach’s alpha of 0.87, indicating strong internal consistency. This supports that the items within our COVID-19 distress scale consistently measure a similar construct. Although initial evidence supports the validity of our scale, further validation with larger, more diverse samples will be essential to establish its robustness and generalizability.

Data were analyzed using SPSS Statistics for Windows, version 16.0 (SPSS Inc., Chicago, Illinois, U.S.A.). The primary outcome was the total psychological distress score. A pandemic-specific subscore was also calculated to assess distress directly attributable to the pandemic context. Descriptive statistics, Pearson’s correlation, and One-Way ANOVA were used to test associations between distress scores and demographic or clinical variables.

## 3 Results

The questionnaire garnered responses from 264 participants, including those who were waiting for surgery or had already undergone surgery but experienced delays due to the COVID-19 pandemic, and who are currently receiving adjuvant treatment. The questionnaire respondents spanned an age range of 18 to 86 years, with a mean age of 54.24 years and a standard deviation (SD) of 14.91 years. Gender distribution was almost equal, with 124 (47%) males and 140 (53%) females. The most common cancer type in the sample was breast cancer (27.7%), followed by genitourinary (18.9%) and gastrointestinal (13.2%) cancers. Musculoskeletal and brain/CNS cancers each accounted for 7.6%, while spine and ophthalmic cancers were least represented (1.1%). All patients in this study had experienced a COVID-19-related delay in their surgical treatment plan. At the time of data collection, just over half had already undergone surgery (54.9%), while others were receiving adjuvant or neoadjuvant chemotherapy (23.95%), were still awaiting surgery (14.0%), or were receiving adjuvant radiotherapy (3.8%), immunotherapy (2.7%), or hormonal therapy (0.8%) (Table 1).

**Table 1:** Demographics of participants.

Baseline characteristics	Level	Frequency	Percentage
Gender	Male	124	47.0%
	Female	140	53.0%
Marital status	Single	24	9.1%
	Married	183	69.3%
	Divorced	4	1.5%
	Widow	53	20.1%
Tumors/malignancies	Breast	79	27.65%
	Genitourinary	50	18.9%
	Gastrointestinal	35	13.2%
	Musculoskeletal	20	7.6%
	Brain and CNS	20	7.6%
	Head and neck	13	4.9%
	Thoracic	10	3.8%
	Ophthalmic	3	1.1%
	Spine	3	1.1%
	Unspecified	12	4.5%
Current type of intervention (at time of data collection)	Scheduled for surgery (currently delayed)	37	14.0%
	Adjuvant and neoadjuvant chemotherapy	63	23.95%
	Underwent surgery	145	54.9%
	Adjuvant radiotherapy	10	3.8%
	Adjuvant immunotherapy	7	2.7%
	Adjuvant hormonal therapy	2	0.8%
Age	Min		18 years
	Max		86 years
	Mean		54.24 years
	SD		14.912

Younger patients exhibited higher distress levels compared to older patients (with an age cutoff value of 54 years, the group’s mean age) (*p* < 0.0001) (Table 2). Conversely, Females reported higher overall distress levels than males (*p* = 0.018), although there were no significant differences in pandemic-related distress between genders (based on items 21, 24–27 in the questionnaire) (Table 3). Never-married individuals demonstrated higher distress levels compared to others (*p*-value = 0.008) (Table 4). A strong positive correlation was observed between total distress scores and pandemic-related questions scores (*R* = 0.691, *p* < 0.0001) (Table 5). Notably, participants diagnosed with brain and central nervous system (CNS) tumors exhibited higher distress levels compared to those with other malignancies (*p* = 0.004) (Table 6).

**Table 2:** Mean age cutoff value in correlation with total stress score.

Age groups	Mean	*N*	Std. deviation	*p* value
Below 54	30.3600	125	15.32834	**<0.0001**
Above 54	23.8129	139	12.73280	
Total	26.9129	264	14.37269	

Bold values indicate statistically significant results, defined as *p* < 0.05.

**Table 3:** Correlation between gender, total stress score and pandemic-specific questions subscore.

Gender	Mean	*N*	Std. Deviation	*p* value
Total stress score				
Male	0.6332	124	0.36143	
Female	0.7405	140	0.36869	**0.018**
Total	0.6901	264	0.36853	
Pandemic-specific questions sub-score				
Male	0.9306	124	0.58158	
Female	0.9400	140	0.53089	0.891
Total	0.9356	264	0.55423	

Bold values indicate statistically significant results, defined as *p* < 0.05.

**Table 4:** Correlation between marital status (MS) and total stress score.

Marital status	Mean	N	Std. deviation	*p* value
Total stress score				
Never-married	0.9156	24	0.32561	**0.008**
Married	0.6576	183	0.38301	
Divorced	0.4936	4	0.10954	
Widow	0.7150	53	0.30838	
Total	0.6901	264	0.36853	

Bold values indicate statistically significant results, defined as *p* < 0.05.

**Table 5:** Correlation table.

Variables compared	*R*^2^	*p*
Correlation table—Pearson		
Mean stress score—mean pandemic-specific questions	0.69	<0.0001
Mean stress score—age (yrs.)	−0.127	0.04
Mean pandemic-specific questions—age (yrs.)	−0.023	0.705

**Table 6:** Correlation between total stress score and type of malignancy.

Malignancies	Mean	N	Std. deviation	*p* value
Total stress score				
Genitourinary	0.5195	50	0.28097	
Bone musculoskeletal	0.6372	20	0.32713	
Brain and CNS	0.7859	20	0.50227	**0.004**
Breast	0.7433	79	0.36291	
Gastrointestinal	0.7260	35	0.31248	
Total	0.6792	204	0.35944	

Bold values indicate statistically significant results, defined as *p* < 0.05.

Significant differences were noted in total stress scores and pandemic-related questions subscore between participants awaiting treatment (current delayed group) and other groups (0.8718 vs. 0.6605, *p* < 0.0001/1.3135 vs. 0.8740, *p* = 0.001) (Table 7). In general, respondents reported higher levels of distress in pandemic-specific questions (32.3%) than in general distress levels (14%) (Table 8).

**Table 7:** Correlation between patients waiting for surgery (delay) vs. other interventions, total stress score and pandemic-related questions sub-score.

Intervention		Total stress score	Total stress score mean
Waiting for surgery (current delay)	Score	34.0000	0.8718
	*N*	37	37
	Std. deviation	11.06797	0.28379
Others	Score	25.7577	0.6605
	*N*	227	227
	Std. deviation	14.53675	0.37274
Total	Score	26.9129	0.6901
	*N*	264	264
	Std. deviation	14.37269	0.36853
*P* value		**0.001**	**0.001**

Intervention		Pandemic-related sub-score	Pandemic-related sub-score mean
Waiting for surgery (current delay)	Score	6.5676	1.3135
	*N*	37	37
	Std. deviation	2.15433	0.43087
Others	Score	4.3700	0.8740
	*N*	227	227
	Std. deviation	2.74177	0.54835
Total	Score	4.6780	0.9356
	*N*	264	264
	Std. deviation	2.77114	0.55423
*P* value		**<0.0001**	**<0.0001**

Bold values indicate statistically significant results, defined as *p* < 0.05.

**Table 8:** Percentage of each answer to all questions vs. pandemic-specific questions.

Level of stress	Total questionnaire (39/39)	Pandemic-specific questions (5/39)
Always	14%	32.3%
Sometimes	40%	29%
Never	46%	38.7%

## 4 Discussion

This study highlights the significant psychological impact of COVID-19-related surgical delays on oncology patients at a tertiary cancer center in Jordan. Distress was more pronounced among younger patients, females, never-married individuals, and those still awaiting surgery. Patients with brain/CNS tumors showed the highest distress levels. Notably, pandemic-specific concerns contributed more to overall distress than general anxiety or depression symptoms. During the pandemic, institutional safety protocols were implemented at KHCC, including telemedicine and restricted visitation. While necessary, such measures may have contributed to increased psychological strain, emphasizing the importance of assessing distress among this vulnerable population.

These findings are particularly relevant in the context of low- and middle-income countries, which faced amplified psychological challenges during the pandemic due to limited healthcare resources and systemic vulnerabilities (Sigdel et al., 2020; Kazmi et al., 2020; Othman, 2020). Oncology patients, already at increased risk for nosocomial infections such as COVID-19, consistently reported distress levels exceeding those typically observed in this population (Miaskowski et al., 2020; Kamboj and Sepkowitz, 2009; Yu et al., 2020).

Our analysis revealed that 14% experienced persistent distress, 40% experienced occasional distress, and 46% reported no complaints. When considering COVID-19-related issues, 145 individuals reported significant distress, with 48.9% feeling distressed upon hearing coronavirus-related news. This aligns with previous studies showing that frequent exposure to COVID-19 information, particularly through unverified or sensationalized media, was strongly associated with heightened anxiety and distress (Moghanibashi-Mansourieh, 2020; Zhou et al., 2020). Similar patterns have been reported globally, where the “infodemic” of overwhelming and often unreliable information contributed substantially to psychological burden during the early stages of the pandemic (Chen et al., 2022; Xu and Liu, 2021). These findings suggest that beyond treatment delays, information overload and uncertainty served as key drivers of distress among cancer patients—highlighting the need for reliable, transparent communication strategies during health crises.

Conversely, 165 individuals did not associate the pandemic with their treatment plans, and 113 did not experience restlessness due to treatment delays. Miaskowski et al. observed increased levels of sleep disturbances, fatigue, cognitive impairment, and pain among oncology patients due to COVID-19, irrespective of its impact on treatment plans (Yu et al., 2020).

Our analysis revealed that unmarried individuals—singles, divorced, and widowed—were more susceptible to distress than married individuals, likely reflecting the protective effect of social companionship (Banet, 1978). Additionally, participants under 54 years exhibited significantly higher distress levels than older patients. This finding aligns with previous research indicating that younger adults with cancer or chronic illnesses often report greater psychological distress compared to older adults (Goulia et al., 2012; Martins-Klein et al., 2021). Explanations include younger patients’ increased exposure to technology and social media, which, while facilitating information dissemination during the pandemic, have been associated with greater anxiety and poorer psychological functioning (Holman et al., 2019). Furthermore, older adults may engage more frequently in spontaneous emotion regulation strategies that mitigate distress (Goulia et al., 2012).

Andersen et al. emphasized that timely psychological interventions can substantially improve overall health outcomes in cancer patients (Andersen et al., 2007). Nonetheless, a persistent gap remains between the prevalence of mental health disorders and those seeking treatment among oncology populations (Holland and Alici, 2010). Historical data from previous pandemics show an increase in patients presenting with psychological distress (McDonnell et al., 2012), underscoring the urgent need for health systems to address these concerns proactively. Given the inadequacy of many global healthcare structures in managing pandemic-associated mental health challenges, early identification and intervention—ranging from cognitive-behavioral therapy to pharmacological treatment—are critical to preventing progression to more severe psychiatric conditions. Psychological distress has also been linked to poorer cancer-related outcomes, including increased mortality (Sanderson et al., 2020; Hamer et al., 2009). In line with this, KHCC has implemented referral pathways to provide psychological counseling for patients identified with elevated distress levels, aiming to deliver reassurance and tailor treatment plans accordingly.

Our study has limitations. It was conducted during a specific period of the ongoing pandemic, potentially limiting generalizability. The self-administered questionnaire did not allow for comprehensive psychological assessments typically conducted in a hospital setting. A follow-up study is warranted to assess changes in responses among the same population. Additionally, future research could apply validated cut-off scores to categorize distress levels, which may facilitate clearer group comparisons and enhance the practical translation of findings into clinical screening and support tools.

## 5 Conclusion

In conclusion, our findings highlight significant disparities in distress levels among oncology patients during the COVID-19 pandemic. Younger individuals, particularly those under 54 years of age, and female participants exhibited heightened levels of distress. Patients awaiting (delayed group) treatment and those diagnosed with central nervous system cancer, irrespective of treatment status, reported the highest stress levels. Additionally, COVID-19-specific inquiries revealed elevated stress levels. These observations emphasize the need for tailored interventions and support systems, particularly for vulnerable demographics, to address and alleviate psychological distress effectively.

## Data Availability

The raw data supporting the conclusions of this article will be made available by the authors, without undue reservation.
