# Supplementary material for: Understanding psychological distress affecting surgical oncology patients during the COVID-19 pandemic—experience from a tertiary cancer care center in the Middle East
Source: Front Psychol. 2025 Oct 30;16:1427835. doi: 10.3389/fpsyg.2025.1427835 (PMC12611941; doi:10.3389/fpsyg.2025.1427835)
Supplement: Supplementary file 1 [file Supplementary_file_1.docx]

**Appendix A: Arabic Questionnaire**

دراسة أثر الضيق النفسي على مرضى السرطان خلال جائحة فايروس كورونا - تجربة مركز الحسين للسرطان

عزيزي المشارك ، نظرا للظروف الصحية التي يمر بها العالم أجمع و أردننا الحبيب أيضا من تفشي وباء كورونا ونظرا للقوانين التي تم إصدارها الملزمة بحظر التجول، نحن في مركز الحسين للسرطان نعمل جاهدين لعودة الرعاية الطبية كما كانت عليه من ذي قبل.

لذلك فأنت مدعو/ة للمشاركة في هذه الدراسة للباحث الدكتور محمود المصري وفريقه البحثي والتي تهدف إلى اكتشاف مستويات الضيق النفسي الذي قد تكون تعرضت له خلال الفترة الماضية وهي فترة حظر التجول خلال انتشار وباء كورونا، كما ان هذه الدراسة ستساعدنا في تّعرف إذا كانت الاجراءات الطبية التي تم اتباعها مثل تأجيل بعض العمليات الجراحة قد كان لها أثر على مستويات الضيق النفسي لديك ام لا.

إذا وافقت/ي على المشاركة في الدراسة الحالية سوف يطلب منك تعبئة استبيانة الضيق النفسي وصفحة المعلومات الديموغرافية المرفقة، من المتوقع ان يستغرق ملئ هذه الاستبانة (25) دقيقة كحد أقصى، علماً بإن العدد المتوقع ان يشارك بالدراسة هو(350) مريض ومريضة.

وسوف تحفظ هذه البيانات في ملف خاص على الجهاز الخاص بالباحث الرئيسي فقط في مركز الحسين للسرطان لحين الانتهاء من الدراسة، و لن يتطبع عليها الا فريق البحث وقد تطلع عليها اللجنة المؤسسية في مركز الحسين للسرطان.

لا يوجد اي مخاطر جسدية متعلقة بمشاركتك في هذه الدراسة، ومن غير المتوقع أن تشعر باي انزعاج نفسي ايضاً ولكن في حال حدث ذلك فيمكنك التوقف عن المشاركة في الدراسة إذا رغبت بذلك، كما سيتم تحويلك للأخصائية النفسية لتقديم الدعم النفسي المناسب .

المشاركة في هذه الدراسة طوعية، يحق لك الانسحاب من المشاركة في أي وقت خلال الدراسة ولن يؤثر ذلك بأي شكل من الاشكال على الرعاية الطبية المقدمة لك.

لا توجد فوائد مباشرة تتعلق بمشاركتك في هذه الدراسة، ولكن قد تسهم مشاركتك في زيادة فهمنا لمستويات الضيق التي قد تعرضت لها خلال الفترة الماضية مما قد يساعدنا في تحسين نوعية الخدمات الطبية والنفسية المقدمة في مركز الحسين للسرطان.

شاكرين لك تعاونكم

في حال رغبتك في معرفة اي معلومات اضافية حول الدراسة يمكنك التواصل مع الباحث الرئيسي الدكتور محمود المصري

هاتف رقم : 079556992

تم مراجعة هذه الدراسة من قبل اللجنة المؤسسية في مركز الحسين للسرطان ، هاتف : 065300460 فرعي : 1669

البريد الالكتروني: [irboffice@khcc.jo](mailto:irboffice@khcc.jo)

*** البيانات الديموغرافية :**

العمر :

الجنس :□ ذكر □ أنثى

الحالة الاجتماعية :

□ أعزب/عزباء □متزوج/ة □ مطلق/ة □أرمل/ة □منفصل/ة

عدد الابناء :

الدخل الشهري :

□أقل من 500 دينار أردني

□500 من 1000 دينار أردني .

□أكثر من 1000 دينار أردني .

تغطية العلاج :

□صناديق الخير / مؤسسة الحسين للسرطان

□الديوان الملكي

□ تأمين خاص

□النفقة الخاصة / Chash

□ برنامج رعاية

□ وزارة الصحة

□غير ذلك

التشخيص الطبي :

**استبانة الضيق النفسي**

**الرجاء قراءة العبارات التالية وأختيار ما ينطبق عليك او ظهر لديك خلال فترة حظر التجول منذ تاريخ 21/3/2020 فقط:**

**إذ يمثل الخيار "دائماً" إلى أنك توافق على العبارة و تنطبق عليك بصفة قوية ودائمة.**

**إذ يمثل الخيار "أحياناً" إلى أنك توافق على العبارة و تنطبق عليك بعض الأحيان ولا تنطبق عليك أحيانا أخرى .**

**إذ يمثل الخيار "إبدا" إلى أنك لا توافق على العبارة ولا تنطبق عليك أبداً .**

| **ابدأ** | **أحيانا** | **دائما** | **العبارة** | **الرقم** |
| --- | --- | --- | --- | --- |
|  |  |  | لا أستطيع الجلوس براحة واسترخاء |  |
|  |  |  | أنا حساس أكثر من الآخرين |  |
|  |  |  | لا أتطلع للمستقبل بسعادة وأمل |  |
|  |  |  | أنا مشغول في التفكير بمخاوفي من المستقبل |  |
|  |  |  | أعتقد أني أكثر عصبية من الآخرين |  |
|  |  |  | لا استمتع بوجود أهلي وأحبتي من حولي خلال هذه الفترة |  |
|  |  |  | أعاني كل عدة ليالي من الكوابيس المزعجة |  |
|  |  |  | عادة لا أكون هادئا وأي شيء يستثيرني |  |
|  |  |  | تمر بي فترات من التوتر لا أستطيع الجلوس طويلاً |  |
|  |  |  | أشعر بالقلق دون مبرر |  |
|  |  |  | لدي شعور غير مريح كما لو أن الأسوأ لم يأتي بعد |  |
|  |  |  | كثيرا أشعر بالتوتر لدرجة أعجز عن النوم هذا السؤال متشابه مع السؤال رقم 4 |  |
|  |  |  | لا استمتع بالقراءة أو التلفزيون |  |
|  |  |  | لا اشعر بالهدوء والاستقرار |  |
|  |  |  | أتمنى أن اكون سعيدا مثل الاخرين |  |
|  |  |  | كثيرا ما أشعر بأني سوف أنفجر من الضيق والضجر |  |
|  |  |  | الحياة بالنسبة لي تعب ومضايقات |  |
|  |  |  | تنقصني الثقة بالنفس |  |
|  |  |  | لا اشعر بالارتياح وانه سوف يحدث لي شيء سيء |  |
|  |  |  | أبكي بسهولة |  |
|  |  |  | أتأثر كثيرا بالأحداث الحالية التي تخص وباء كورونا |  |
|  |  |  | لا أستطيع التركيز في شيء واحد |  |
|  |  |  | أنا غالبا أحلم بأشياء من الأفضل ألا أخبر أحد بها |  |
|  |  |  | مخاوفي كثيرة جد ا بالمقارنة مع أفراد أسرتي فيما يخص وضعي الصحي |  |
|  |  |  | دائما ينتابني شعور بالقلق عندما استمع للأخبار المتعلقة وباء كورونا |  |
|  |  |  | أنا لا أثق في نجاح علاجي بعد الآن |  |
|  |  |  | انتظار العلاج يجعلني عصبي جدا |  |
|  |  |  | كثيرا ما أشعر أن قلبي يخفق بسرعة |  |
|  |  |  | أعاني من الآلام بالمعدة |  |
|  |  |  | أعاني دوخة وإغماء |  |
|  |  |  | أتضايق من الصداع، وجع الرقبة و وجع الظهر |  |
|  |  |  | نومي مضطرب ومتقطع |  |
|  |  |  | أعاني كثيرا من الصداع |  |
|  |  |  | تصيبني نوبات من الغثيان |  |
|  |  |  | أشعر بالتثاقل، البطء و قلة الهمة |  |
|  |  |  | كثيرا جدا ألاحظ أن يداي ترتعش عندما أقوم بأي عمل |  |
|  |  |  | أعاني من إسهال شديد |  |
|  |  |  | يداي وقدماي باردتان في العادة |  |
|  |  |  | يحصل لي حالات إمساك تضايقني |  |

إنتهت الإستبانة

مع أمنياتنا لكم بدوام الصحة والعافية
