# Supplementary material for: Understanding psychological distress affecting surgical oncology patients during the COVID-19 pandemic—experience from a tertiary cancer care center in the Middle East
Source: Front Psychol. 2025 Oct 30;16:1427835. doi: 10.3389/fpsyg.2025.1427835 (PMC12611941; doi:10.3389/fpsyg.2025.1427835)
Supplement: Supplementary file 2 [file Supplementary_file_2.docx]

**Appendix B: English questionnaire**

**Understanding Psychological Distress Affecting Surgical Oncology Patients during the COVID-19 Pandemic – KHCC Experience**

**Psychological Distress in Surgical Oncology Patients**

Dear Participant,

Due to the global health conditions and the situation in our beloved Jordan, particularly the spread of the coronavirus pandemic and the issuance of mandatory curfew regulations, the King Hussein Cancer Center is diligently working towards resuming medical care to its pre-pandemic standards.

Therefore, you are invited to participate in this study led by Dr. Mahmoud El-Masri and his research team. The study aims to explore the levels of psychological distress you may have experienced during the recent period of curfew imposed during the spread of the coronavirus. Additionally, the study will help us understand if medical measures, such as the postponement of certain surgical procedures, have had an impact on your levels of psychological distress.

If you agree to participate in the current study, you will be asked to complete the Psychological Distress Questionnaire and the attached Demographic Information page. It is expected that filling out this questionnaire will take a maximum of 25 minutes. The anticipated number of participants in the study is 350 patients.

Your data will be kept in a secure file on the main researcher's device only at the King Hussein Cancer Center until the study is completed. Only the research team will have access to it, and the Institutional Review Board at the King Hussein Cancer Center may review it.

There are no physical risks associated with your participation in this study, and it is not expected that you will experience any psychological discomfort. However, if you do, you have the option to withdraw from the study at any time. In such a case, you will be referred to a mental health professional for appropriate support.

Participation in this study is voluntary, and you have the right to withdraw at any time during the study without affecting the medical care provided to you.

There are no direct benefits associated with your participation in this study. However, your participation may contribute to our understanding of the levels of distress you have experienced during the recent period, potentially helping us improve the quality of medical and psychological services provided at the King Hussein Cancer Center.

Thank you for your cooperation.

If you would like additional information about the study, you can contact the principal researcher, Dr. Mahmoud El-Masri, at the following phone number: 079556992.

This study has been reviewed by the Institutional Review Board at the King Hussein Cancer Center. You can contact them at 065300460, extension 1669, or via email at [irboffice@khcc.jo](mailto:irboffice@khcc.jo)

**Demographic Information:**

Age______________________

Gender:

□ Male

□ Female

Marital Status:

□ Single

□ Married

□ Divorced

□ Widowed

□ Separated

Number of Children:­­­­­­­­­­­­­­_______________

Monthly Income:

□ Less than 500 Jordanian Dinars

□ 500 to 1000 Jordanian Dinars

□ More than 1000 Jordanian Dinars

Medical Coverage:

□ Charity organizations / King Hussein Cancer Foundation

□ Royal Court

□ Private Insurance

□ Private Expenses / Cash

□ Care Program

□ Ministry of Health

□ Other

Medical Diagnosis: ____________________

Psychological Distress Questionnaire:

Please read the following statements and choose the option that applies to you or has occurred to you during the curfew period since March 21, 2020 only. The options are as follows:

- "Always" indicates strong and consistent agreement with the statement.

- "Sometimes" indicates occasional agreement with the statement, with occasional disagreement.

- "Never" indicates no agreement with the statement at all.

| Numb | Statement | Always | Sometimes | Never |
| --- | --- | --- | --- | --- |
| 1. | I cannot sit comfortably and relax |  |  |  |
| 2. | I am more sensitive than others |  |  |  |
| 3. | I do not look forward to the future with happiness and hope |  |  |  |
| 4. | I am preoccupied with thoughts of future fears |  |  |  |
| 5. | I believe I am more nervous than others |  |  |  |
| 6. | I do not enjoy the presence of my family and loved ones around me during this period |  |  |  |
| 7. | I suffer from disturbing nightmares every few nights |  |  |  |
| 8. | I am usually not calm, and anything easily upsets me |  |  |  |
| 9. | I go through periods of tension where I cannot sit for long |  |  |  |
| 10. | I feel anxious without justification |  |  |  |
| 11. | I have an uncomfortable feeling as if the worst is yet to come |  |  |  |
| 12. | Often, I feel so tense that I am unable to sleep. This question is similar to question number 4 |  |  |  |
| 13. | I do not enjoy reading or watching TV |  |  |  |
| 14. | I do not feel calm and stable |  |  |  |
| 15. | I wish I could be as happy as others |  |  |  |
| 16. | Often, I feel like I will explode from stress and boredom |  |  |  |
| 17. | Life, for me, is fatigue and annoyance |  |  |  |
| 18. | I lack self-confidence |  |  |  |
| 19. | I do not feel at ease, and I expect something bad to happen to me |  |  |  |
| 20. | I cry easily |  |  |  |
| 21. | I am greatly affected by current events related to the coronavirus pandemic |  |  |  |
| 22. | I cannot concentrate on one thing |  |  |  |
| 23. | I often dream of things I'd rather not tell anyone about |  |  |  |
| 24. | My fears about my health are much greater than those of my family members |  |  |  |
| 25. | I always feel anxious when listening to news about the coronavirus |  |  |  |
| 26. | I no longer trust in the success of my treatment |  |  |  |
| 27. | Waiting for treatment makes me very nervous |  |  |  |
| 28. | Often, I feel that my heart beats rapidly |  |  |  |
| 29. | I suffer from stomach pains |  |  |  |
| 30. | I experience dizziness and fainting |  |  |  |
| 31. | I am bothered by headaches, neck pain, and back pain |  |  |  |
| 32. | My sleep is disturbed and interrupted |  |  |  |
| 33. | I often suffer from headaches |  |  |  |
| 34. | I have bouts of nausea |  |  |  |
| 35. | I feel sluggish, slow, and lack motivation |  |  |  |
| 36. | Very often, I notice that my hands tremble when I do any work |  |  |  |
| 37. | I suffer from severe diarrhea |  |  |  |
| 38. | My hands and feet are usually cold |  |  |  |
| 39. | I experience episodes of constipation that bother me |  |  |  |

The questionnaire has ended. Wishing you good health and well-being.
